# Associations of attention-deficit/hyperactivity disorder with inflammatory diseases. Results from the nationwide German Health Interview and Examination Survey for Children and Adolescents (KiGGS)

**DOI:** 10.1007/s40211-023-00479-8

**Published:** 2023-08-17

**Authors:** Lena Boemanns, Julia Staab, Thomas Meyer

**Affiliations:** 1 Klinik für Psychosomatische Medizin und Psychotherapie, Universität Göttingen, Göttingen, Germany; 2 Department of Psychosomatic Medicine and Psychotherapy, German Center for Cardiovascular Research, University of Göttingen, Von-Siebold-Str. 5, Göttingen, Germany

**Keywords:** 25-Hydroxyvitamin D, Atopic dermatitis, Otitis media, Herpes simplex, Inflammation, 25-Hydroxyvitamin D, Atopische Dermatitis, Otitis media, Herpes simplex, Entzündung

## Abstract

**Background:**

Despite conflicting data, some studies have suggested a pathophysiological relationship between inflammation and attention-deficit/hyperactivity disorder (ADHD).

**Methods:**

Using data from the nationwide and representative German Health Interview and Examination Survey for Children and Adolescents (KiGGS; *n* = 6922 study participants aged 11–17 years), this post hoc analysis assessed the associations between ADHD and three common inflammatory diseases.

**Results:**

Results showed univariate associations between ADHD and lifetime inflammatory diseases including atopic dermatitis (*p* = 0.002), otitis media (*p* = 0.001), and herpes simplex infection (*p* = 0.032). In logistic regression models adjusted for clinically relevant confounders, we found that ADHD remained a significant predictor of all three inflammatory diseases (atopic dermatitis, Exp(β) = 1.672, 95% confidence interval [CI] 1.239–2.257, *p* = 0.001; otitis media, Exp(β) = 1.571, 95% CI 1.209–2.040, *p* = 0.001; herpes simplex, Exp(β) = 1.483, 95% CI 1.137–1.933, *p* = 0.004).

**Conclusion:**

Our findings demonstrate a positive link between ADHD and peripheral inflammatory diseases, including atopic dermatitis, otitis media, and herpes simplex infection. Further studies are needed to understand the exact pathophysiological mechanisms underlying these associations.

## Abstract (de)

**Zielsetzung:**

Trotz widersprüchlicher Daten haben einige Studien auf einen pathophysiologischen Zusammenhang zwischen Entzündungen und Aufmerksamkeitsdefizit‑/Hyperaktivitätsstörung (ADHS) hingewiesen.

**Methoden:**

Anhand von Daten aus dem bundesweiten und repräsentativen Kinder- und Jugendgesundheitssurvey (KiGGS) wurden in dieser Post-hoc-Analyse an *n* = 6922 Studienteilnehmerinnen und Studienteilnehmern im Alter von 11 bis 17 Jahren die Zusammenhänge zwischen ADHS und drei häufigen entzündlichen Erkrankungen untersucht.

**Resultate:**

Die Ergebnisse zeigten univariate Assoziationen zwischen ADHS und peripheren entzündlichen Erkrankungen einschließlich atopischer Dermatitis (*p* = 0,002), Otitis media (*p* = 0,001) und Herpes-simplex-Infektion (*p* = 0,032). In logistischen Regressionsmodellen, die auf klinisch relevante Störfaktoren adjustiert wurden, fanden wir, dass ADHS ein signifikanter Prädiktor für alle drei entzündlichen Erkrankungen blieb (atopische Dermatitis, Exp(β) = 1,672, 95 % Konfidenzinitervall [KI] = 1,239–2,257; *p* = 0,001; Otitis media, Exp(β) = 1,571, 95 % KI = 1,209–2,040; *p* = 0,001; Herpes simplex, Exp(β) = 1,483, 95 % KI = 1,137–1,933; *p* = 0,004).

**Schlussfolgerung:**

Unsere Ergebnisse zeigen einen positiven Zusammenhang zwischen ADHS und peripheren entzündlichen Erkrankungen, einschließlich atopischer Dermatitis, Otitis media und Herpes-simplex-Infektion. Weitere Studien sind erforderlich, um die genauen pathophysiologischen Mechanismen zu verstehen, die diesen Zusammenhängen zugrunde liegen.

## Introduction

With a worldwide prevalence of approximately 5% in children and adolescents, attention-deficit/hyperactivity disorder (ADHD) is a common and frequently studied neurodevelopmental condition; however, its etiology and pathophysiology are not yet fully understood [1]. Some studies have hypothesized that ADHD can be considered a nonallergic hypersensitivity disorder [2, 3], and efforts have been made to decipher the underlying mechanisms linking the onset or presence of ADHD symptoms to altered immune reactions [4–6]. Using immune markers and antioxidant components in blood samples from untreated ADHD patients and a healthy control group, Verlaet et al. demonstrated that there may indeed be a link between immune system disorders and the occurrence of ADHD [7]. In a case–control study, Darwish et al. found that serum interleukin‑6 (IL-6) levels were significantly higher in ADHD patients (*n* = 60) compared with a healthy control group [8]. This finding was later confirmed by Elsadek et al., although the IL‑6 levels did not correlate with ADHD symptom severity [9, 10]. In a previous study from the nationwide and representative German Child and Adolescent Health Survey (KiGGS), we reported significant and inverse associations between the prevalence of ADHD and both serum vitamin D concentration and systolic blood pressure [11]. The well-known immunomodulatory effects of vitamin D may have an impact not only on the prevalence and course of autoimmune and/or infectious diseases, but also on the pathogenesis of ADHD [11, 12].

Based on these observations, the aim of this post hoc study was to assess possible associations between ADHD and the lifetime prevalence of inflammatory diseases, including atopic dermatitis, otitis media, and herpes simplex virus infections, taking into account vitamin D levels and systolic blood pressure recordings. These three diseases were selected because their combination has not been studied before and reliable data on their prevalences were available from the KiGGS data file. In this analysis, we hypothesized that, in subjects with ADHD, there is a comorbidity with inflammatory diseases.

## Methods

### Data collection

For this post hoc study, the subsample of 11- to 17-year-old children and adolescents was selected from the German KiGGS study, because both self- and parent-reported data for this age group were available. The survey, conducted by the Robert Koch Institute from May 2003 to May 2006, involved 17,641 children (8985 boys and 8656 girls) aged 0 to 17 years. The study design has been described in detail elsewhere [13]. The recruitment of participants was accomplished in two steps. First, 167 communities were selected as sampling points all over Germany and, in a second step, participants were randomly chosen from official local registers and invited to participate in the survey. The study included questionnaires completed by parents and adolescents, physical examinations and a computer-assisted personal interview [14]. The study was funded by the German Federal Ministry of Health (BMG) and the German Federal Ministry of Education and Research (BMBF) [15]. All study participants and their legal guardians gave written informed consent. Ethical approval for this study was obtained from the Ethics Committee of the Charité Universitätsmedizin Berlin and additionally from the German Federal Office for Data Protection.

### Clinical assessment

The dataset used for the present analysis included information on sex, age, body mass index (BMI), socioeconomic status, migration status, systolic blood pressure, serum 25-hydroxyvitamin D [25(OH)VitD] concentration, and lifetime prevalences of atopic dermatitis, otitis media, and herpes simplex infection. Following a standardized protocol, body height was measured by trained personnel with an accuracy of 0.1 cm. Participants’ body weight was determined in underwear on a calibrated scale with an accuracy of 0.1 kg. Body mass index was calculated by dividing body weight in kilograms by the square of measured height in meters [16]. Socioeconomic status (SES) was determined based on parental information about participants’ schooling, professional qualifications, net income, and job-related positions for all household members, as recommended by the German Society of Epidemiology [17]. A participant was defined as having a migration background if one or both parents were not born in Germany and/or did not have German citizenship [18]. Blood pressure was measured noninvasively using the Datascope Accuratorr Plus blood pressure monitor and a portable monitor (SOMA Technology, Bloomfield, CT, USA) [19]. Two independent measurements were taken 5 min apart for each subject. For the measurement of 25(OH)VitD, peripheral venous blood was drawn by a pediatrician. Serum was obtained by allowing the blood sample to stand at room temperature for 45 min to clot. Subsequently, the samples were centrifuged and the serum was stored at −50 °C [20]. The determination of 25(OH)VitD concentrations was performed in a special laboratory of the Robert Koch Institute [21]. A change of method for the quantitative determination of 25(OH)VitD was necessary due to stability problems of the analyte, and the fully automated LIAISON chemiluminescence immunoassay (CLIA, DiaSorin, Dietzenbach, Germany) was used [22, 23]. To determine the lifetime prevalence of ADHD, parents were required to answer the question, “Has your child ever been diagnosed with attention-deficit/hyperactivity disorder?” Responses were rated ‘yes’, ‘no’, or ‘I don’t know’ on a three-point scale. If the answer was ‘yes’, the question was expanded to include the additional question, ‘If yes, how was the disorder diagnosed?’; possible responses were ‘medical doctor’, ‘psychologist’, or ‘other’ [24].

Lifetime prevalence of atopic dermatitis, otitis media, and herpes simplex was also assessed using the self-administered questionnaire in which parents reported diseases diagnosed by physicians [25].

### Statistical analysis

Data were extracted from the Robert Koch Institute’s publicly available file and transferred to a computerized database. Statistical analyses were performed using Statistical Package for the Social Sciences version 26 software (IBM, Armonk, NY, USA). A weighting factor was applied to account for unequal sampling probabilities due to differences between respondents and nonrespondents, such as age, sex, and residence in urban or rural areas. To characterize the study population, continuous data are presented using standard deviations and means, whereas categorical data are presented as frequencies and percentages. The chi-square test was used for numeric variables to detect significant differences between groups. Student’s t test was performed for continuous values. To test whether inflammatory diseases were associated with ADHD, a series of logistic regression models were calculated using atopic dermatitis, otitis media, or herpes simplex infection as dependent variables and a similar set of confounding factors (age, sex, BMI, socioeconomic status, migration status, 25(OH)VitD concentration, mean systolic blood pressure, and ADHD). Variables were included as confounders in the regression models, if there was a statistically significant difference between the affected and non-affected groups in at least one of the three univariate comparisons. Given the exploratory nature of this analysis, a Bonferroni correction was not applied. For all tests, statistical significance was defined as *p* < 0.05.

## Results

### Characterization of the study cohort

From the total KiGGS study population of 17,641 children and adolescents, *n* = 6922 participants with available data on ADHD diagnosis were at least 11 years old, and all of these subjects were included in the present post hoc analysis. According to parents’ ratings, 430 of these children and adolescents had been diagnosed with ADHD. The mean age of the participants in the total study population was 14.6 ± 2.0 years, and because of the sampling procedure used in the KiGGS survey, male (49.9%) and female participants were almost equally represented, with a slightly higher proportion of girls. Mean systolic blood pressure and 25(OH)VitD levels were 114.8 ± 10.8 mm Hg and 44.5 ± 27.6 nmol/l, respectively. A lifetime diagnosis of ADHD was reported more frequently in patients with atopic dermatitis (8.1% vs. 5.7%, *p* = 0.002), otitis media (7.1% vs. 5.2%, *p* = 0.001), and herpes simplex (7.3% vs. 5.9%, *p* = 0.032) than in participants without these diseases. Demographic and biochemical characteristics, including participants with and without these conditions, are summarized in detail in Table 1.

**Table 1:** Descriptive statistics of the study cohort. Data are presented as mean values with standard deviations or percentages

	Total study population (*n* = 6922)	Atopic dermatitis		*p* value	Otitis media		*p* value	Herpes simplex		*p* value
		Yes (*n* = 1164)	No (*n* = 5466)		Yes (*n* = 3714)	No (*n* = 2966)		Yes (*n* = 1895)	No (*n* = 4341)	
Age (years)	14.6 ± 2.00	14.5 ± 2.0	14.6 ± 2.0	0.164	14.6 ± 2.0	14.7 ± 2.0	0.074	14.7 ± 2.0	14.6 ± 2.0	0.006
Sex (male, %)	49.9	46.2	50.7	0.005	49.3	50.7	0.274	45.4	51.6	< 0.001
BMI (kg/m^2^)	21.1 ± 4.0	21.0 ± 3.9	21.1 ± 4.1	0.256	21.2 ± 4.1	20.9 ± 4.0	0.018	21.2 ± 4.0	21.0 ± 4.0	0.059
SES	11.7 ± 4.3	12.2 ± 4.3	11.6 ± 4.3	< 0.001	11.9 ± 4.3	11.4 ± 4.4	< 0.001	11.6 ± 4.2	11.8 ± 4.3	0.051
Migrants (%)	11.9	6.4	12.8	< 0.001	7.8	16.7	< 0.001	10.0	11.8	0.004
Mean systolic BP (mm Hg)	114.8 ± 10.8	114.1 ± 10.5	115.0 ± 10.8	0.012	114.7 ± 10.8	114.8 ± 10.9	0.682	114.3 ± 10.5	115.1 ± 11.0	0.006
25(OH)VitD (nmol/l)	44.5 ± 27.6	46.0 ± 25.3	44.1 ± 28.1	0.087	45.1 ± 30.2	43.5 ± 24.0	0.058	47.1 ± 26.6	43.4 ± 28.1	< 0.001
ADHD (%)	6.2	8.1	5.7	0.002	7.1	5.2	0.001	7.3	5.9	0.032

*BMI* body mass index; *BP* blood pressure; *25(OH)VitD* 25-hydroxyvitamin D, *SES* socioeconomic status

**Table 2:** Models using atopic dermatitis, otitis media, or herpes simplex as dependent and ADHD as independent variable adjusted for the indicated confounders

Variable	Exp (B)	95% CI	Wald	*P* value
*Model 1 Atopic dermatitis (R*^*2*^ *=* *0.010, p* *<* *0.001)*				
Age	0.991	0.948–1.036	0.146	0.703
Sex (male)	0.802	0.680–0.945	6.938	0.008
BMI	0.988	0.965–1.011	1.084	0.298
SES	1.028	1.009–1.047	8.210	0.004
Migrant	0.603	0.448–0.811	11.195	0.001
Mean systolic BP	1.000	0.992–1.009	0.004	0.953
25(OH)VitD	1.251	0.927–1.688	2.143	0.143
ADHD	1.692	1.253–2.285	11.775	0.001
*Model 2 Otitis media (R*^*2*^ *=* *0.029, p* *<* *0.001)*				
Age	0.959	0.927–0.992	5.788	0.016
Sex (male)	0.874	0.771–0.990	4.456	0.035
BMI	1.025	1.008–1.043	8.475	0.004
SES	1.032	1.018–1.047	18.715	< 0.001
Migrant	0.434	0.357–0.527	70.977	< 0.001
Mean systolic BP	1.003	0.996–1.009	0.679	0.410
25(OH)VitD	1.025	0.821–1.281	0.048	0.826
ADHD	1.576	1.213–2.048	11.620	0.001
*Model 3 Herpes simplex (R*^*2*^ *=* *0.016, p* *<* *0.001)*				
Age	1.045	1.007–1.084	5.361	0.021
Sex (male)	0.822	0.716–0.944	7.726	0.005
BMI	1.023	1.004–1.042	5.963	0.015
SES	0.982	0.966–0.998	5.049	0.025
Migrant	0.780	0.626–0.973	4.864	0.027
Mean systolic BP	0.989	0.982–0.996	9.297	0.002
25(OH)VitD	1.781	1.375–2.306	18.946	< 0.001
ADHD	1.471	1.129–1.918	8.143	0.004

*BMI* body mass index; *BP* blood pressure; *25(OH)VitD* 25-hydroxyvitamin D, *SES* socioeconomic status, *CI* confidence interval

### Relationship between atopic dermatitis and ADHD

Atopic dermatitis was diagnosed in *n* = 1164 study participants aged 11 years or older, with more probands being female than in the control group (53.8% vs. 49.3%, *p* = 0.005). Further differences between participants with and without atopic dermatitis were found in the socioeconomic status, as participants affected by atopic dermatitis tended to be of higher socioeconomic status (12.2 ± 4.3 vs. 11.6 ± 4.3, *p* < 0.001). Similarly, participants with an immigrant background were less likely to be affected by atopic dermatitis (6.4% vs. 12.8%, *p* < 0.001). In addition, mean arterial blood pressure was lower in the group of participants with atopic dermatitis (114.1 ± 10.5 mm Hg versus 115.0 ± 10.9, *p* = 0.012). A logistic regression model was constructed with atopic dermatitis as the dependent variable and ADHD diagnosis as the independent variable, adjusted for age, sex, BMI, socioeconomic status, migration status, mean systolic blood pressure, and 25(OH)VitD. In the multivariate model, the positive association between atopic dermatitis and ADHD was confirmed (Exp(β) = 1.692, 95% CI = 1.253–2.285, R^2^ = 0.010, *p* = 0.001; Table 2).

### Association between otitis media and ADHD

Subsequently, the cohort was examined with regard to otitis media. In total, *n* = 3714 participants had been diagnosed with otitis media during their lifetime. No significant sex differences were found between participants with and without otitis media (Table 1). However, significant differences were found in socioeconomic status, whereby participants with otitis media more often belonged to a higher socioeconomic status (11.9 ± 4.3 vs. 11.4 ± 4.4, *p* < 0.001). Participants with an immigrant background were less likely to have otitis media, as previously observed for atopic dermatitis (7.8% vs. 16.7%, *p* < 0.001). The results also showed that there was a significant difference in the lifelong prevalence of otitis media in the comparison of the affected group with the unaffected group with regard to ADHD (7.1% vs. 5.2%, *p* = 0.001). A significant positive association between otitis media and the diagnosis of ADHD was confirmed in the corresponding adjusted logistic regression model (Exp(β) = 1.576, 95% CI = 1.213–2.048, R^2^ = 0.029, *p* = 0.001; Table 2).

### Association between herpes simplex and ADHD

A total of *n* = 1895 subjects had a history of a herpes simplex infection, as self-reported by study participants and/or their parents. Compared with the control group, participants diagnosed with herpes simplex were significantly more likely to be female (54.6% vs 48.4%, *p* < 0.001) and less likely to have an immigrant background (10.0% vs 11.8%, *p* = 0.004). Whereas mean arterial blood pressure was lower than in the control group (114.3 ± 10.5 mm Hg vs 115.1 ± 11.0, *p* = 0.006), ADHD was significantly more common in the group of participants with herpes simplex (7.3% vs 5.9%, *p* = 0.032). The results from a correspondingly adjusted logistic regression model confirmed a significant and positive association between a lifelong herpes simplex virus infection and ADHD diagnosis (Exp(β) = 1.471, 95% CI = 1.129–1.918, R^2^ = 0.016, *p* = 0.004). Furthermore, 25(OH)VitD levels were elevated in all three inflammatory diseases, but only in herpes simplex to a significant extent (47.1 ± 26.6 nmol/l vs 43.4 ± 28.1, *p* < 0.001). As expected, this association was also confirmed in the adjusted logistic regression model (Exp(β) = 1.781, 95% CI = 1.375–2.306, *p* < 0.001; Table 2).

## Discussion

In search of a possible association between lifetime prevalence of inflammatory diseases and ADHD, we studied the group of 11- to 17-year-old participants in the KiGGS study. The main finding of our analysis is that clinically reported ADHD was significantly associated with the occurrence of atopic dermatitis, otitis media, and herpes simplex infections in children and adolescents. Binary logistic regression models adjusted for age, sex, BMI, socioeconomic status, migration status, mean systolic blood pressure, and serum 25(OH)VitD level also showed these significant associations. However, we did not find any association between 25(OH)VitD level and the occurrence of atopic dermatitis or otitis media. The models also failed to confirm any association between mean systolic blood pressure and atopic dermatitis or otitis media.

The results of our study are consistent with those of other studies showing a positive association between ADHD and atopic dermatitis. For example, Schmitt et al. demonstrated a positive association between ADHD and atopic dermatitis in a systematic review of 20 studies [26]. Interestingly, no association was found between serum IgE levels and ADHD symptoms in this systematic analysis. In another study, the significant association between ADHD and IgE was also lost after Bonferroni correction [7]. In another systematic review, no significant association was found between the two disorders in two of the six included studies [27].

The results of studies that investigated the association between otitis media and ADHD also show conflicting results. Hagerman and Falkenstein reported that children with learning disabilities who are more likely to have otitis media often also tend to be hyperactive [28], which was replicated in another study [29]. In a cohort of 7578 Danish children, frequent occurrence of otitis media was shown to lead to learning and behavioral problems later in life [30].

There are few data in the literature on the association between herpes simplex virus infection and ADHD or ADHD-type behavioral disorders. Herpes simplex virus 1 seropositivity is associated with various cognitive disorders such as poorer scores on reading and spatial reasoning tests [31] or poorer memory function in adolescents [32]. Similar results were published in another study that measured antibodies to various viruses and found an association with ADHD. It was found that various viral infections could be associated with ADHD-typical behavior [33]. Jørgensen et al. compared a group of healthy children and a group of child psychiatric patients using ELISA to detect herpes simplex virus antibodies, but no overall difference was found between the two groups [34]. In addition, Lin and colleagues reported no significant differences between the patients with herpes simplex infections of the brain and the control group in the occurrence of ADHD [35].

There are several mechanisms that could explain a comorbidity of inflammation and the development of ADHD. Several studies have already shown that IL‑6 and IL-1β are associated with ADHD and ADHD-like symptoms. For example, a follow-up study in neonates showed that children with elevated IL‑6 levels exhibited more frequently attention deficits and aggressive behavior and that children with elevated IL-1β levels had poorer motor skills [36]. In an animal model equivalent to ADHD, spontaneously hypertensive rats were shown to have elevated serum and tissue levels of IL-1β compared to control animals [37]. IL-1β is thought to be involved in microglial activation found in psychiatric disorders, but it has not been conclusively determined whether microglial activation may also be the cause of psychiatric disorders [38].

The main causes of atopic dermatitis are thought to be skin barrier dysfunction due to decreased filaggrin expression, as well as a dysregulated immune response and IgE autoreactivity [39], while otitis media and herpes simplex are both diseases with infectious genesis, either bacterial or viral [40, 41]. In addition, it is already known that children suffering from ADHD show lower distancing behaviors compared to healthy controls [42], which may explain the more frequent occurrence of infectious diseases such as otitis media and herpes simplex.

Due to several limitations of our study, such as the cross-sectional and post hoc design, a causal interpretation for the observed association between ADHD and peripheral inflammatory diseases is not allowed, as the level of evidence does not reach that of a randomized controlled trial. In addition, neither was the ADHD diagnosis formally confirmed by the trained personnel, who performed the clinical examinations, nor were the inflammatory conditions. Although the effect sizes are rather small, the large sample size reveals weaker associations, which may be of interest for the interaction between ADHD and peripheral inflammatory diseases. Another important weakness is that the explained variance is rather small. However, in addition to these limitations, our study has unique strengths, which lie in the fact that it deals with a large cohort of older children and adolescents representative of the German population. Furthermore, information was collected using standardized written questionnaires by well-trained personnel with pediatric experience who were blinded as to the identity of the subjects.

## Conclusion

We found a statistically significant association between peripheral inflammatory disease and the presence of ADHD. To our knowledge, this is the first study to examine an association between ADHD and the three different diseases of atopic dermatitis, otitis media, and herpes simplex infection using the same cohort. Moreover, these associations remained stable when all known relevant confounders were included in the adjusted models. However, further research is needed to unravel the physiological mechanisms underlying these associations.

## Abbreviations

ADHD: Attention-deficit/hyperactivity disorder
BMI: Body mass index
KiGGS: German Child and Adolescent Health Survey
25(OH)VitD: 25-Hydroxyvitamin D
